# I Knew You Weren’t Going to Like Me! Neural Response to Accurately Predicting Rejection Is Associated With Anxiety and Depression

**DOI:** 10.3389/fnbeh.2019.00219

**Published:** 2019-10-02

**Authors:** Megan E. Quarmley, Brady D. Nelson, Tessa Clarkson, Lauren K. White, Johanna M. Jarcho

**Affiliations:** ^1^Department of Psychology, Temple University, Philadelphia, PA, United States; ^2^Department of Psychology, Stony Brook University, Stony Brook, NY, United States; ^3^Department of Child and Adolescent Psychiatry and Behavioral Science, Children’s Hospital of Philadelphia, Philadelphia, PA, United States

**Keywords:** ventral striatum, social reward, monetary reward, fMRI, anxiety, depression, peer evaluation

## Abstract

Anxiety and depression often emerge in adolescence. A normative increase in the desire for peer acceptance may be one of many contributing factors. These shifts occur during a phase of development in which neural reward networks, including structures such as the ventral striatum, undergo critical changes. Despite the salience of peer feedback during adolescence, neural responses to reward have largely been examined in the monetary domain, leaving many open questions about responses to social rewards. Moreover, most paradigms do not tease apart different aspects of reward processing (e.g., receiving feedback, being correct). Anxiety and depression are also associated with alterations in reward networks; however, little is known about how anxiety and depression in adolescence relate to differences in social vs. non-social reward processing. In this study, adolescents (*n* = 28) underwent fMRI while completing novel monetary and social feedback tasks, which tease apart reward domain (social/monetary), valence (positive/negative), and outcome (correct/incorrect). Participants were shown a pair of stimuli (doors/age-matched peers) and asked to indicate which stimulus would provide positive (win money/social like) or negative (lose money/social dislike) feedback. Participants then received feedback about the purported accuracy of their response. Region-of-interest analyses showed that left ventral striatum response varied by domain (social/monetary), valence (positive/negative), and outcome (correct/incorrect) of reward. Additionally, unique associations between anxiety, depression, and brain function were observed for correct, but not for incorrect trials, in the social, but not monetary task. Specifically, adolescents with high anxiety symptoms, but low depression, displayed greater left ventral striatum activation when correctly identifying peers who gave dislike (vs. like) feedback. Thus, anxious youth exhibited enhanced activation in a brain region implicated in reward processing when they accurately predicted someone was going to dislike them. Higher levels of both depression and anxiety symptoms were associated with greater striatal activation to correctly identifying peers who gave like (vs. dislike) feedback. These results suggest a neural mechanism by which negative prediction biases may be reinforced in anxious youth.

## Introduction

The importance of peer relationships increases during adolescence as the brain undergoes changes in neural networks critical for processing reward (Nelson and Guyer, 2011). This network is composed of interconnected brain regions implicated in reward sensitivity, such as the striatum, orbitofrontal cortex and anterior cingulate cortex (Galvan, 2010; Richards et al., 2013), substantia nigra, and the ventral tegmental area (VTA; Haber and Knutson, 2010; Wang et al., 2016), as well as regions involved in self-regulation in rewarding contexts, such as the prefrontal cortex (Galvan, 2010; Richards et al., 2013; Wang et al., 2016). However, extensive human and animal studies have identified the dopamine-rich ventral striatum as a critical hub in this network (Galvan, 2010; Richards et al., 2013; Wang et al., 2016). Although social acceptance is a powerful reward for adolescents (Guyer et al., 2012), neural response to reward has largely been examined in the monetary domain. Testing reward processing in the social domain may be particularly important when considering the neural mechanisms that promote symptoms of anxiety (Beesdo-Baum et al., 2012) and depression (Thapar et al., 2012). These symptoms increase dramatically in adolescence and are associated with alterations in reward-related brain function (Kujawa et al., 2018). Although social stressors often potentiate symptoms of anxiety and depression, direct tests of the association between symptoms and neural responses across reward domains are rare. Moreover, most research examining relations between brain function and reward processing have confounded the intrinsically rewarding experience of being correct (Satterthwaite et al., 2012) with positively valenced outcomes (Rademacher et al., 2010; Meuwese et al., 2018). Yet, symptoms of adolescent anxiety and depression may be differentially associated with dysregulated processing of intrinsic (being correct) and extrinsic (receiving a positively valenced outcome) rewards across social and non-social domains. We tested these relations in adolescents with a range of anxiety and depression symptoms by implementing novel, well-matched fMRI tasks that disentangle the brain’s response to the intrinsic reward of being correct from its response to positively and negatively valenced outcomes in social and non-social (i.e., monetary) domains.

Reward processing is commonly conceptualized as a uniform construct in which incentives elicit equivalent neural and behavioral responses regardless of domain (e.g., social, monetary; Ethridge et al., 2017). The few studies that have contrasted reward in social and monetary domains using fMRI have used monetary and social reward tasks with markedly different experimental designs (Delgado et al., 2008; Izuma et al., 2008; Wake and Izuma, 2017) or tasks in which the subjective value of monetary and social rewards differ (Rademacher et al., 2010). For example, Izuma et al. (2008) measured the relation between monetary and social reward by contrasting neural response during a monetary gambling task with neural response to reading positive self-descriptors (Izuma et al., 2008), while Delgado et al. (2008) utilized a solitary monetary bidding task and a social competition monetary bidding task. Despite the paucity of well-matched tasks, studies in adults demonstrate that the ventral striatum is engaged by both social and monetary rewards (Delgado et al., 2008; Izuma et al., 2008; Rademacher et al., 2010; Wake and Izuma, 2017). However, whether there are differences in the magnitude of ventral striatum activation to social and monetary rewards remains inconsistent in the literature. For example, while Delgado et al. (2008) found differences in right ventral striatum activation between conditions, Izuma et al. (2008) found no difference in ventral striatum activation between their social and monetary reward tasks. Thus, it is possible that the disparity in these findings could be due to methodological differences in the tasks themselves. Given that reward sensitivity (Ernst and Spear, 2009) and desire for social acceptance peak in adolescence, it is critical to delineate social vs. non-social reward processing during this developmental period.

Even fewer studies have sought to tease apart neural response to intrinsic and extrinsic rewards. Being correct is an intrinsically rewarding experience (Satterthwaite et al., 2012) that engages the ventral striatum in the absence of incentives or performance feedback (Han et al., 2010; Wolf et al., 2011). Although this effect is most pronounced during adolescence (Satterthwaite et al., 2012), most prior studies examining brain function during reward processing in adolescence fail to disentangle neural response to choosing correct outcomes (intrinsic reward) from winning money for having chosen those outcomes (extrinsic reward). Therefore, it is unclear whether prior findings that demonstrate adolescents have heightened ventral striatal engagement to rewards reflect a sensitivity to intrinsic or extrinsic rewards.

Prior fMRI studies of reward processing have also linked alterations in striatal activation to depression (Silk et al., 2014; Telzer et al., 2014) and anxiety (Guyer et al., 2006; Bar-Haim et al., 2009; Lago et al., 2017). Depression is associated with a blunted neural response to both social (Monk et al., 2008; Olino et al., 2015) and monetary (Gotlib et al., 2010; Sharp et al., 2014; Weinberg et al., 2015; Nelson et al., 2016) rewards in adults and children, whereas anxiety is associated with enhanced neural response to social (Guyer et al., 2012; Spielberg et al., 2015) and monetary (Bar-Haim et al., 2009) rewards. We recently conducted an EEG study in young adults in which we examined relations between depression and the magnitude of the reward positivity (RewP), an event-related potential that indexes engagement of the reward system (Distefano et al., 2018), in response to social (being liked) and monetary (winning money) rewards. While both social and monetary rewards elicited the RewP, more severe symptoms of depression were associated with a blunted RewP to social, but not monetary, rewards. Specifically, women with more severe depressive symptoms had a blunted RewP in response to being liked by same-sex peers. This suggests that there are unique relations between depression and neural response to social, but not monetary, reward. However, given the poor spatial resolution of EEG, it is unclear whether the blunted RewP reflects diminished engagement in the ventral striatum. Moreover, extant work has not directly contrasted response to social and monetary rewards in individuals with a range of both depression and anxiety symptoms; thus, the interplay of symptoms of anxiety and depression on the brain’s response to social vs. non-social reward, particularly in adolescents, remains unclear.

While our prior EEG study provides promising evidence for the relationship between depression and social reward, distinguishing neural response to receiving positively valenced social outcomes from the intrinsic experience of being correct was not tested. Given that alterations in brain regions implicated in reward processing are linked to symptoms of anxiety (Guyer et al., 2006; Bar-Haim et al., 2009) and depression (Silk et al., 2014; Telzer et al., 2014), it is critical to determine if these alterations are specific to intrinsic or extrinsic reward processing. Moreover, individuals with anxiety and depression often exhibit negative predictions about social outcomes (Beck et al., 1979; Clark and Wells, 1995; Joiner and Coyne, 1999; Smith et al., 2018). Given these biases and the role that social stressors often play in triggering symptoms of depression and anxiety, it is critical to test if relations between symptoms and brain function differ by reward domain.

In the present study, we used fMRI to isolate differences in ventral striatal response to correctly or incorrectly predicting positive and negative feedback in both social and monetary domains in adolescents. Well-matched social and monetary paradigms were employed to disentangle neural responses to positively valenced outcomes from the intrinsic reward of being correct. Additionally, we examined these neural responses in relation to anxiety and depression symptoms. We focus on ventral striatum because prior studies consistently find ventral striatum activation in response to monetary and social reward. We hypothesized that ventral striatal response to outcomes (correct or incorrect) would differ by reward valence (positive or negative) across reward domains (social or monetary). We also hypothesized that given the salience of peers to adolescents, altered neural response to social, but not monetary, reward would be associated with anxiety and depressive symptoms.

## Materials and Methods

### Participants

Participants were adolescents (*n* = 37; females = 18) aged 11–15 (*M* = 13.32; *SD* = 1.28) who were free of psychotropic medication and had no contraindications for fMRI. Informed written parental consent and written participant assent were obtained prior to participation, and all procedures were approved by the Institutional Review Board at Stony Brook University and were conducted in accordance with the Helsinki Declaration.

### Measures

Depression was measured using the Children’s Depression Inventory (CDI; Kovacs, 1992), a 27-item self-report measure of depressive symptoms in school-aged children and adolescents. Anxiety was measured using the Screen for Child Anxiety Related Emotional Disorders (SCARED; Birmaher et al., 1999), a 41-item self-report questionnaire that assesses severity of anxiety symptoms in youth aged 8–18. The self-report version of this measure was used because it has greater sensitivity for detecting symptoms of anxiety than parent-report (Rappaport et al., 2017).

### Procedure

Prior to the experimental session, participants were told they were completing a social evaluation study and were asked to submit a digital picture of themselves that would be sent to other purported participants their age across the country. Participants believed that these peers would receive a text message asking them to view the photo and indicate whether they thought they would “like” or “dislike” the participant. The picture would then disappear after 5 min. At the beginning of the experimental session, participants were told that they would be asked to guess which peers “liked” or “disliked” them and that they would also be completing a monetary guessing task. Participants completed self-report questionnaires and underwent mock scanning to gain familiarity with the MRI environment. Participants then underwent fMRI while completing the monetary and social reward tasks in a counterbalanced order. At the end of the session, participants responded to questions about their experience with the task to ensure they were engaged and believed the credibility of the peer feedback. Nearly all (*n* = 35; 94%) participants had high levels of task engagement and believed they were receiving feedback from actual peers. Participants were then debriefed.

#### fMRI-Based Monetary and Social Reward Tasks

The monetary and social reward tasks were administered using Eprime software [“Psychology Software Tools Inc., 2016; (E-Prime 2.0). Retrieved from http://www.pstnet.com”]. There were four conditions (monetary win, monetary loss, social like, and social dislike) that were presented in a counterbalanced order. Each condition included 30 trials. Each task was completed across two, 4.55-min runs. Each run included two blocks: one block of monetary win or social like trials, and one block of monetary loss or social dislike trials (15 trials per block). Trials were separated by a variable duration intertrial interval (1,100–11,600 ms; *M* = 3,500 ms).

##### Monetary Reward Task (Figure 1)

At the beginning of each block, participants were informed if the block contained monetary win trials or monetary loss trials. In monetary win blocks, participants were instructed to choose the door behind which there was a $0.25 prize. In monetary loss blocks, participants were instructed to choose the door behind which there was a $0.25 monetary loss. Each trial began with the presentation of two identical doors (3,000 ms). Participants then used a button box to select either the left or right door on the screen. After stimulus offset, a fixation cross was presented for 600 ms before participants received feedback about the accuracy of their choice (1,000 ms). Participants were told that there were three possible scenarios for each monetary win/loss trial: (1) both doors contained a $0.25 monetary win/loss; (2) one door contained a $0.25 monetary win/loss while the other door resulted in a break-even outcome (i.e., neither win nor loss); or (3) both doors resulted in a break-even outcome. This ensured that the feedback the participant received would only be informative about the door they chose and not the door they *did not* choose. For example, if a participant chose a door and received feedback indicating a break-even result, the other door could have been a win/loss door (consistent with trial scenario above) or it could have been another break-even door (consistent with trial scenario above). In monetary win trials, feedback was either a green arrow pointing upward (↑) meaning the participant correctly selected the monetary win door, or a white horizontal dash (-), which indicated incorrect selection of the break-even door, resulting in no monetary win. In monetary loss trials, correct selection of the monetary loss door was indicated by a red arrow pointing downward (↓), while incorrect selection of the break-even door resulting in no monetary loss was indicated by a white horizontal dash (-).

**Figure 1:**
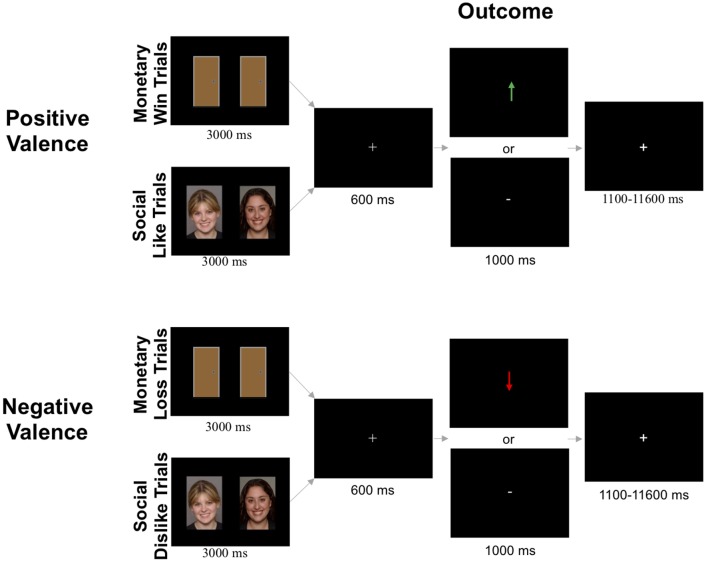
Schematic for fMRI-based social and monetary reward paradigms. Images displayed are taken from a database where written informed consent is not required.

##### Social Reward Task (Figure 1)

The social like and dislike tasks were identical to the monetary win and loss tasks, respectively, except pictures of gender-matched peers (i.e., two female faces or two male faces) were presented instead of doors. The social reward task consisted of 120 images of age-matched peers compiled from multiple sources [National Institute of Mental Health’s Child Emotional Faces picture set (Egger et al., 2011) and internet databases of non-copyrighted images]. The pictures of purported peers had positive facial expressions, were cropped so that individuals were pictured from their shoulders up, and were edited to have an identical solid gray background. Smiling faces were used because they are common in social reward tasks (Richards et al., 2013; Jarcho et al., 2015; Distefano et al., 2018), and are subject to less misinterpretation than neutral faces (Rapee and Heimberg, 1997; Davis et al., 2016). Images were constrained to a standard size (2.75 inch width × 4 inch height). There were an equal number of trials with male and female peers across the social like and dislike conditions (30 pairs each, 60 total).

At the beginning of each block of trials, participants were informed if the block contained social like trials or social dislike trials. In social like blocks, participants were instructed to choose the peer that liked them. In social dislike blocks, they were instructed to choose the peer that disliked them. Participants were told that there were three possible situations for each trial: (1) both people said they would like/dislike the participant; (2) one person said they would like/dislike the participant while the other person never rated the participant; or (3) neither person rated the participant. In social like trials, correct selection of the person who said they would like the participant was indicated by a green arrow pointing upward (↑). In social dislike trials, correct selection of the person who said they would dislike the participant was indicated by a red arrow pointing downward (↓). In both social like and dislike conditions, incorrect selection of the person who never rated the participant was indicated by a white horizontal dash (-).

### fMRI Acquisition

Functional images were acquired using a 3T Siemens PRISMA MRI scanner. Blood Oxygenation Level-Dependent (BOLD) sensitive functional images were acquired using a gradient echo-planar imaging (EPI) sequence (224 mm in FOV, TR = 2,100 ms, TE = 23 ms, voxel size of 2.3 × 2.3 × 3.5 mm^3^, flip angle = 83°, interleaved slice acquisition). Each run included 37 functional volumes. To facilitate anatomical localization and coregistration of functional data, a high-resolution structural scan was acquired (sagittal plane) with a T1-weighted magnetization-prepared rapid acquisition gradient echo (MPRAGE) sequence (250 mm in FOV, TR = 1,900 ms, TE = 2.53 ms, voxel size of 1.0 × 1.0 × 1.0 mm^3^, flip angle = 9°).

### fMRI Preprocessing and Individual Level Analysis

Preprocessing and fMRI analyses were conducted using AFNI (Cox, 1996). Standard pre-processing steps were implemented with afni_proc.py; these steps included slice timing, coregistration, smoothing to 6-mm full-width half maximum (FWHM), spatial normalizing to standard Talairach space, and resampling, which resulted in 2-mm^3^ voxels. Task-specific events (spanning the duration of each event) were modeled using a block function. An additional six regressors modeled motion residuals. Temporally adjacent repetition times (TRs) with a Euclidean-norm motion derivative greater than 1 mm were omitted from the model *via* censoring. Individual-level fMRI data were manually reviewed and subjects were excluded for motion and signal drop-out (*n* = 9), resulting in a final sample of 28 individuals. Results remained consistent when non-deceived participants were removed (*n* = 2). To retain power, these individuals were included in the final analyses.

**Figure 2:**
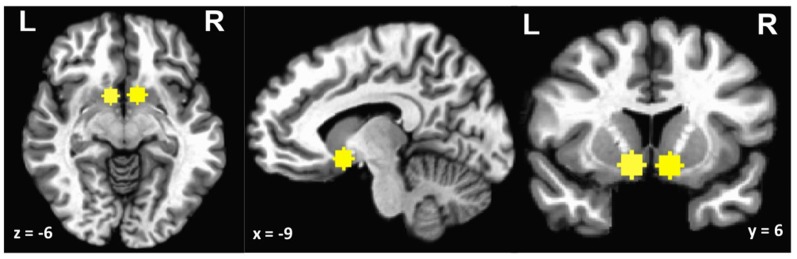
Ventral striatum region of interest (ROI).

Based on *a priori* hypotheses, we performed a region of interest (ROI) analysis on the ventral striatum. Ventral striatum was defined in MNI space using Neurosynth (Yarkoni et al., 2011). Left and right ventral striatum ROIs were derived using the meta-analytic search term “ventral striatum” (415 studies). Because a portion of the full cluster extended into ventricle and white matter, 6-mm sphere masks were created around central voxels (MNI left *x* = −9, *y* = 6, *z* = −6; right *x* = 9, *y* = 6, *z* = 6; see Figure 2). Individual-level data from these ROI masks were then extracted for each subject.

### Data Analysis

Group level analyses were conducted in IBM SPSS Statistics, Version 25.0 (“Mac SPSS Statistics for Windows,” IBM Corp, 2017). To investigate task effects, we conducted a Domain (monetary, social) × Valence (positive: monetary win/social like, negative: monetary loss/social dislike) × Outcome (correct, incorrect) analysis of variance (ANOVA). Next, to examine relations between the neural response to reward processing and anxiety and depression symptoms, we conducted a Domain (monetary, social) × Valence (positive: monetary win/social like, negative: monetary loss/social dislike) × Outcome (correct, incorrect) ANCOVA with depression and anxiety symptoms included as continuous covariates of interest. Decomposition analyses were performed for significant interactions related to task effects and *a priori* hypotheses regarding relations between ventral striatum response to reward domain, valence, and outcome to anxiety and depression.

## Results

**Figure 3:**
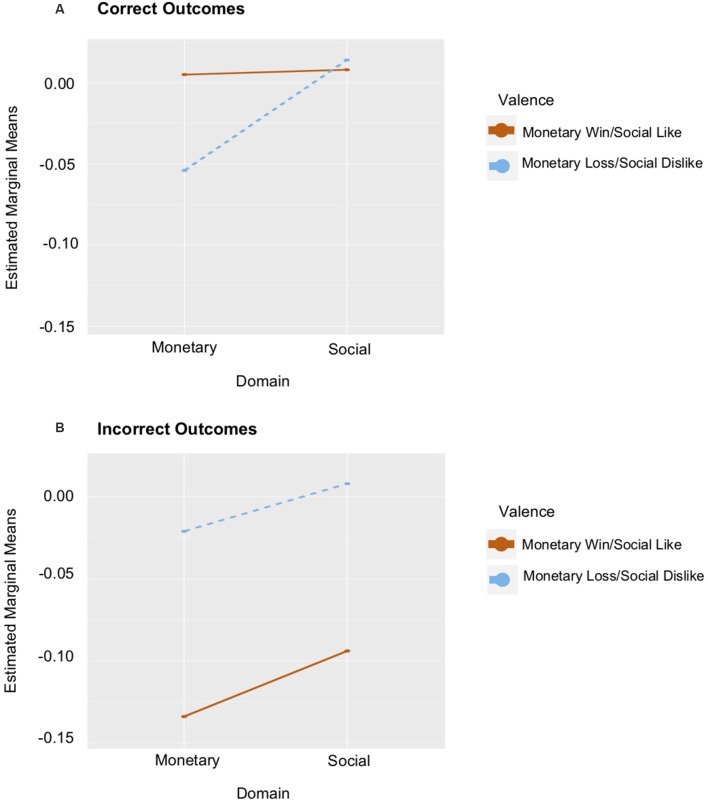
Graphs of ventral striatum response to Domain × Valence controlling for Anxiety and Depression. **(A)** The red line depicts estimated marginal means of the monetary win and social like conditions for correct outcome trials. The dashed blue line depicts the estimated marginal means of the monetary loss and social dislike conditions for correct outcome trials. **(B)** The same relations are depicted for incorrect outcomes.

A test of task effects demonstrated that while there was no Domain × Valence × Outcome interaction, a Valence × Outcome interaction emerged in the left ventral striatum (*F*_(1,27)_ = 15.937, *p* < 0.001, $\eta_{\text{p}}^{2}$ = 0.371). This interaction was driven by greater left ventral striatum response to correctly guessing positive outcomes (*M* = 0.0295), than to incorrectly guessing positive outcomes (*M* = −0.103; *t*_(27)_ = 4.819, *p* < 0.001). There was no significant difference between correctly (*M* = −0.022) and incorrectly (*M* = −0.005) guessing negative outcomes (*t*_(27)_ = 0.633, *p* = 0.532). There was also a main effect of Outcome (*F*_(1,27)_ = 8.464, *p* = 0.007, $\eta_{\text{p}}^{2}$ = 0.239), such that there was greater left ventral striatum response to correctly (*M* = 0.004), relative to incorrectly (*M* = −0.054) guessing outcomes (*t*_(27)_ = 2.909, *p* = 0.007). When anxiety and depression were included as covariates, a more complex task effect emerged. Specifically, a Domain × Valence × Outcome interaction was observed (*F*_(1,24)_ = 5.064, *p* = 0.034, $\eta_{\text{p}}^{2}$ = 0.174; see Figure 3). A significant interaction emerged for correct trials (*F*_(1,24)_ = 4.303, *p* = 0.049, $\eta_{\text{p}}^{2}$ = 0.152), but not for incorrect trials (*F*_(1,24)_ = 1.642, *p* = 0.212, $\eta_{\text{p}}^{2}$ = 0.064). Although not significant, these effects were more prominent in the monetary domain.

The hypothesized Domain × Valence × Outcome × Anxiety × Depression interaction also emerged (*F*_(1,24)_ = 5.043, *p* = 0.034, $\eta_{\text{p}}^{2}$ = 0.174). The significant five-way interaction is decomposed in the below sections. Task effects with and without depression and anxiety were not observed in right ventral striatum. See Table 1 for other main and interaction effects that do not directly relate to our* a priori* hypotheses.

**Table 1:** Results for the left ventral striatum Domain × Valence × Outcome × Anxiety × Depression ANCOVA.

Main effects	*F*	*p*	$\eta_{\text{p}}^{2}$
Domain	0.166	0.687	0.007
Valence	0.483	0.494	0.020
Outcome	4.289	0.049^a^	0.152
**Interaction Effects**			
Domain × Valence	0.390	0.538	0.016
Domain × Outcome	0.072	0.790	0.003
Valence × Outcome	4.551	0.043^b^	0.159
Domain × Valence × Outcome	5.064	0.034^c^	0.174
Domain × Anxiety × Depression	0.000	0.998	0.000
Valence × Anxiety × Depression	1.430	0.243	0.056
Outcome × Anxiety × Depression	0.175	0.680	0.007
Domain × Valence × Anxiety × Depression	1.978	0.172	0.076
Domain × Outcome × Anxiety × Depression	0.007	0.935	0.000
Valence × Outcome × Anxiety × Depression	1.430	0.243	0.056
Domain × Valence × Outcome ×	5.043	0.034	0.174
Anxiety × Depression

*^a^Correct Outcomes (M = −0.007; SE = 0.022); Incorrect Outcomes (M = −0.060; SE = 0.019). ^b^Monetary Win/Social Like Correct Outcomes (*M* = 0.006; *SE* = 0.030); Monetary Win/Social Like Incorrect Outcomes (*M* = −0.114; *SE* = 0.028); Monetary Loss/Social Dislike Correct Outcomes (*M* = −0.020; *SE* = 0.029); Monetary Loss/Social Dislike Incorrect Outcomes (*M* = −0.006; *SE* = 0.025). ^c^Social Like Correct Outcomes (*M* = 0.008; *SE* = 0.048); Social Like Incorrect Outcomes (*M* = −0.094; *SE* = 0.056); Social Dislike Correct Outcomes (*M* = 0.014; *SE* = 0.045); Social Dislike Incorrect Outcomes (*M* = 0.008; *SE* = 0.037); Monetary Win Correct Outcomes (*M* = 0.005; *SE* = 0.035); Monetary Win Incorrect Outcomes (*M* = −0.134; *SE* = 0.038); Monetary Loss Correct Outcomes (*M* = −0.054; *SE* = 0.039); Monetary Loss Incorrect Outcomes (*M* = −0.021; *SE* = 0.031)*.

### Do Domain × Valence Effects Vary for Correct and Incorrect Outcomes Depending on Anxiety and Depression?

To determine if the interactive effects of domain, valence, and symptoms on brain function varied by outcome, we conducted two separate Domain (monetary, social) × Valence (monetary win/social like, monetary loss/social dislike) × Depression × Anxiety ANCOVAs, one for correct outcomes and one for incorrect outcomes. A significant interaction emerged for correct trials (*F*_(1,24)_ = 7.195, *p* = 0.013, $\eta_{\text{p}}^{2}$ = 0.231), but not for incorrect trials (*F*_(1,24)_ = 0.192, *p* = 0.665, $\eta_{\text{p}}^{2}$ = 0.008). Furthermore, there was a significant Domain × Valence interaction in correct (*F*_(1,24)_ = 4.303, *p* = 0.049, $\eta_{\text{p}}^{2}$ = 0.152), but not incorrect trials (*F*_(1,24)_ = 0.990, *p* = 0.330, $\eta_{\text{p}}^{2}$ = 0.040). Thus, further decomposition analyses focused on correct outcomes.

### For Correct Outcomes, Do Valence Effects Vary by Domain Depending on Anxiety and Depression?

To determine if interaction effects for correct outcomes were specific to the domain of the reward (i.e., monetary or social), we next conducted two separate Valence (monetary win/monetary loss, social like/social dislike) × Depression × Anxiety ANCOVAs, one for correct trials in the social domain and one for correct trials in the monetary domain. For the social task, results indicated a Valence × Anxiety × Depression interaction (*F*_(1,24)_ = 8.577, *p* = 0.007, $\eta_{\text{p}}^{2}$ = 0.263). However, these effects were not found in the monetary task (*F*_(1,24)_ = 0.566, *p* = 0.459, $\eta_{\text{p}}^{2}$ = 0.023). Thus, the left ventral striatum differentially responds to social valence type (i.e., like vs. dislike) when an adolescent is correct, but activation varies based on severity of anxiety and depression symptoms.

### For Correct Outcomes in the Social Domain, Do Valence Effects Differentially Relate to Anxiety and Depression?

**Figure 4:**
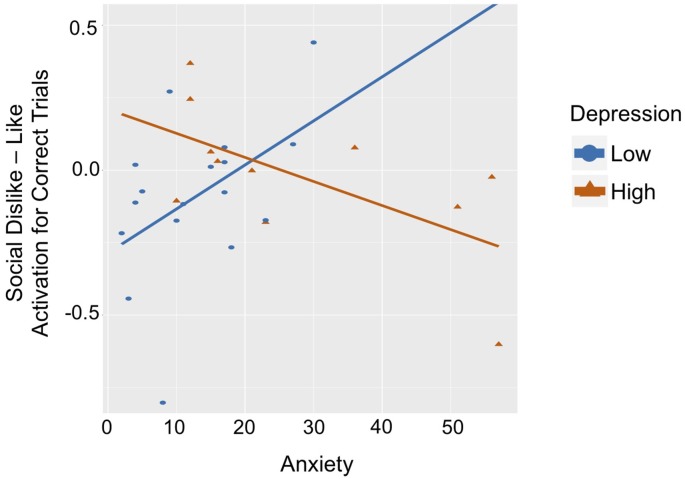
Graph of correlation between ventral striatum response to Social Dislike–Like Correct Outcome Trials and anxiety for the low-depression group (blue line) and high-depression group (red line).

While statistical analyses utilized fully dimensional measures, to facilitate the interpretation of this complex interaction and for illustrative purposes, participants were binned into low and high depression groups using a median split (low < 9; high ≥ 9 on the CDI). See Table 2 for group characteristics. Social Like and Dislike trials were also contrasted (dislike–like) for ease of interpretation (see Figure 4). In the low depression group (*n* = 17), there was a positive correlation between anxiety and ventral striatum activation to correct outcomes in dislike as compared to like trials (*r* = 0.472, *p* = 0.056). Specifically, among youth with *low* levels of depression, *more severe* anxiety symptoms were associated with *greater* *activation* in the striatum when participants learned they had correctly guessed that a peer *disliked* (vs. liked) them. The opposite relation was observed in the *high* depression group (*n* = 11; *r* = −0.617, *p* = 0.043). Specifically, among youth with higher levels of depression, *more severe* anxiety symptoms were associated with *greater activation* in the striatum to correctly guessing that a peer *liked* (vs. disliked) them. Furthermore, the association between brain activation and anxiety in the low-depression group was significantly different from this relation in the high-depression group (Fisher’s *r* to *z* = 2.78, *p* = 0.005).

**Table 2:** Characteristics of low and high depression groups used for illustrative purposes in decomposition analyses.

Characteristic	Low depression	High depression	(*n* = 17)	(*n* = 11)
Gender		
Female (*n*)	5	8
Male (*n*)	12	3
Age *M* (SD)	13.41 (1.33)	13.18 (1.25)
SCARED total anxiety *M* (SD)	12.94 (8.53)	28.09 (18.55)
CDI total depression *M* (SD)	4.29 (2.37)	15.91 (5.26)

## Discussion

To our knowledge, this is the first study to utilize well-matched social and monetary reward paradigms to disentangle ventral striatal response to reward domain, valence, and outcome in adolescence. Importantly, the study design enabled us to tease apart striatal response to the intrinsic reward of being correct from the valence of social and monetary outcomes. Furthermore, we examined how depression and anxiety symptoms were associated with adolescents’ striatal response in this paradigm. We found that activation in the left ventral striatum exhibited unique associations with symptoms of anxiety and depression depending on valence outcome, when receiving correctly predicted social feedback. These results support the idea that reward processing mechanisms are not uniform, but sensitive to contextual factors related to incentives. This sensitization may, in turn, be influenced by individual differences in anxiety and depression symptoms.

Considering task-based effects without the influence of symptoms of anxiety and depression revealed greater left ventral striatal response to correctly relative to incorrectly guessing outcomes. Thus, consistent with prior reports (Wolf et al., 2011; Satterthwaite et al., 2012), the intrinsic reward of being right engaged a critical hub in an appetitive processing circuit. However, this pattern of engagement was valence specific; greater activity was observed for positive, but not negative outcomes. These results underscore the importance of utilizing tasks that are sensitive to both the valence of appetitive outcomes and the intrinsic reward of being correct.

Interestingly, a more complex pattern of task effects emerged in the model controlling for anxiety and depression symptoms. The striatum responded differently to feedback depending on its domain (social/monetary), valence (positive/negative), and outcome (correct/incorrect). Specifically, when participants learned that they guessed correctly, there were differences in the striatal response depending on reward domain and valence. The same relation was not observed when participants learned that they guessed incorrectly. These effects were predominantly found in the monetary task, such that ventral striatum activation was greater to correctly guessing positively rather than negatively valenced outcomes. Surprisingly, similar relations were not observed in the social task. These findings are consistent with research demonstrating that the ventral striatum is closely linked to processing appetitive outcome and is engaged by being correct (Han et al., 2010; Wolf et al., 2011; Satterthwaite et al., 2012). Our findings support and extend this work by illustrating that ventral striatum activation to the intrinsically rewarding experience of being correct may also influence the way that other characteristics of reward, such as reward domain and valence, are processed.

Another unique feature of this study is that we contrast positively valenced outcomes and negatively valenced outcomes each with a null social condition (i.e., did not rate). Thus, we are able to examine the unique relation that each condition has with neural reward responsivity. Notably, this aim differs from most prior studies that directly contrast positive and negative social outcomes and are unable to tease apart unique effects for each condition. Therefore, prior work examining social reward in adolescence has not been able to disentangle ventral striatum response to positive (relative to null) vs. negative (relative to null) peer feedback. Our findings illustrate important differences in striatal function to the different feedback conditions. Specifically, the ventral striatum activates more to correctly guessing positive than negative monetary rewards; this pattern did not emerge for social rewards. Overall, these results highlight the importance of studying relations between neural activation to social rewards and of directly comparing reward domains.

We also found unique associations between anxiety and depression symptoms and ventral striatum activation to correctly guessing social outcomes. Among adolescents with low depressive symptoms, more severe anxiety was associated with greater striatal activation to correctly guessing if a peer *disliked* (vs. liked) them. Prior literature has shown that, separately, anxiety and depression are associated with altered neural responses to reward in different ways. For example, greater anxiety symptoms have been associated with an enhanced neural response to reward (Bar-Haim et al., 2009), an effect that we also found. However, these studies did not examine if relations were specific to social or monetary rewards, or intrinsic or extrinsic rewards. Our findings, therefore, extend prior work by showing that greater anxiety symptoms were associated with increased ventral striatum activation to correctly guessing about a negative social outcome. Predicting that social interactions will have a negative outcome is a common feature of anxiety (Clark and Wells, 1995; Smith et al., 2018). Therefore, our findings suggest that individuals with anxiety may find it rewarding to confirm their negative predictions about social experiences. These results may shed light on a possible mechanism by which negative social biases are reinforced and maintained. This is important because one of the central tenets of Cognitive Behavioral Therapy, the prevailing psychological treatment for anxiety (Chambless and Gillis, 1993), is to identify and change negative predictions, such as those about social outcomes (Hofmann, 2007). Therefore, elucidating neural mechanisms that underlie the reinforcement of these negative prediction biases may inform targets for interventions.

Conversely, among participants with high depressive symptoms, more severe anxiety was associated with greater striatal activation to correctly guessing if a peer *liked* (vs. disliked) them. Many studies have shown that depression is associated with a blunting of neural responsivity to reward (Landes et al., 2018). For example, our prior EEG study found that more severe symptoms of depression were associated with decreased RewP to social feedback (Distefano et al., 2018). These inconsistencies may be related to task-based features, but could also reflect an interplay between anxiety and depressive symptoms. Specifically, the brain’s response to reward may vary depending on the level of symptom comorbidity. Thus, while depression and anxiety are both associated with negative prediction biases about social interactions (Beck et al., 1979; Clark and Wells, 1995; Joiner and Coyne, 1999; Smith et al., 2018), the neural mechanisms underlying the concurrent maintenance of these symptoms may be distinct.

Despite its strengths, this study is not without limitations. First, results need to be replicated in a larger sample. Moreover, because of the small sample size, we were unable to test for effects of participant and peer gender on brain function. Prior work has identified important sex differences in brain-based sensitivity to reward (Distefano et al., 2018; Greimel et al., 2018). For example, our prior EEG study found an association between depression and blunted RewP only in female participants when they were responding to same-sex peers. Thus, it is possible that present results may differ for adolescent males and females, or by the gender of the peer giving feedback. This study was also cross-sectional; thus, it is unclear whether altered neural response to social reward results in more symptoms of anxiety and depression or whether the presence of symptoms of anxiety and depression leads to altered neural response to social reward. Studies that leverage longitudinal designs are needed to test the predictive value and stability of neural response patterns to social reward and their relation to symptoms of psychopathology. Furthermore, longitudinal research could determine if relations between neural responses sensitive to reward domain, valence, outcome, and symptoms of psychopathology change across development. Indeed, children exhibit lower neural reward sensitivity than adolescents (Ernst and Spear, 2009), and socially anxious adolescents, but not adults, exhibit increased striatal activity to unexpected positive feedback from high-value peers (Jarcho et al., 2015). Lastly, the participants in this study were from an unselected community sample that had relatively low symptoms of anxiety and depression. It is possible that the association between ventral striatal engagement and symptoms of anxiety and depression may differ with more severe levels of psychopathology. However, the fact that these results emerged even in a subclinical sample suggests that reward to correctly guessing negative social predictions may be instantiated early in the course of a disorder and could promote symptoms.

In sum, this study begins to disentangle the complicated interplay between anxiety, depression, and neural activation to different characteristics of reward during adolescence. Results highlight that reward is not a unified construct. They suggest that engagement of neural mechanisms implicated in reward may depend on reward domain, valence, and the accuracy of the predicted outcome. Prior literature often conflates these different aspects of reward processing; however, results from the current study support the need to disentangle them in future work. Although tentative, our results also underscore complex relations between anxiety and depression and neural responses to reward in adolescence. Both anxiety and depression are associated with negative predictions about social interactions, yet there may be distinct, disorder-specific mechanisms that reinforce these negative predictions. Future work needs to directly connect these neural reward patterns to adolescents’ adaptive and maladaptive behaviors in social interactions, as these relations likely play a critical role in forming strategies for navigating peer relationships. By understanding the mechanisms through which youth with and without psychopathology process different characteristics of reward, we may be able to inform treatment programs at this crucial stage of development.

## Data Availability Statement

The datasets generated for this study are available on request to the corresponding author.

## Ethics Statement

This study was carried out in accordance with the recommendations of the Institutional Review Board of Stony Brook University with written informed consent from all subjects. All subjects gave written informed consent in accordance with the Declaration of Helsinki. The protocol was approved by the Institutional Review Board of Stony Brook University.

## Author Contributions

MQ, JJ and BN contributed to the conception and design of the study. MQ and JJ organized the database. MQ, JJ and LW performed the statistical analysis. MQ wrote the first draft of the manuscript. MQ, JJ and TC wrote sections of the manuscript. All authors contributed to manuscript revision, and read and approved the submitted version.

## Conflict of Interest

The authors declare that the research was conducted in the absence of any commercial or financial relationships that could be construed as a potential conflict of interest.
